# Cell-free DNA screening for sex chromosome aneuploidies by non-invasive prenatal testing in maternal plasma

**DOI:** 10.1186/s13039-020-0478-5

**Published:** 2020-03-12

**Authors:** Yipeng Wang, Shanshan Li, Wei Wang, Yuan Dong, Meng Zhang, Xin Wang, Chenghong Yin

**Affiliations:** Prenatal Diagnostic Center, Beijing Obstetrics and Gynecology Hospital, Capital Medical University, Beijing, 100026 China

## Abstract

**Background:**

Non-invasive prenatal testing (NIPT) has been confirmed as the most accurate screening test for trisomies 21, 18, and 13. However, reports on NIPT performance in sex chromosome aneuploidies (SCA) based on real clinical data are still limited.

**Methods:**

High-throughput massively parallel genomic sequencing (MPS) technique was used to screen for fetal SCAs as part of the research to determine the potential value of NIPT in detecting fetal SCAs in the second trimester. A number of 12,243 consecutive cases from a single center were included in this study.

**Results:**

The positive predictive value (PPV) of NIPT in the present study was 57.6%, which was divided and categorized by individual SCAs as follows: 21.4% for Turner syndrome (45,X), 75.0% for Triple X syndrome (47,XXX), 90.9% for Klinefelter syndrome (47,XXY), and 75.0% for XYY syndrome (47,XYY).

**Conclusion:**

The NIPT-based SCA test cannot be used as a diagnostic method, and performing an invasive confirmation test on NIPT-based SCA-positive cases is strongly recommended.

## Introduction

Prenatal screening of fetal aneuploidy tests, based on sonography and maternal biochemistry findings, which has been focused on trisomy 21 and, recently, on trisomies 18 and 13, has a detection rate within 50–95% at a 5% false-positive rate [1, 2]. Since the introduction of cell-free DNA (cfDNA) testing and massively parallel sequencing (MPS), non-invasive prenatal testing (NIPT) has been confirmed as the most accurate screening test for these aneuploidies [3, 4]. NIPT has been widely applied for screening for trisomies 21, 18, and 13 using cfDNA in maternal plasma [5]. The combined specificity for these autosomal aneuploidies was found to be 99.9% [6].

Sex chromosome aneuploidies (SCA), including monosomy X (45,X), Klinefelter syndrome (47,XXY or 48,XXYY), triple X syndrome (47,XXX), and 47,XYY, with a combined prevalence of 1: 500 are more common than the major trisomies. Although most cases of SCA are generally mild, without intellectual disability, some have a well-established phenotype that can include physical abnormalities, learning delays, and infertility [2, 3, 7]. However, SCA is often found as an accidental discovery following diagnostic ultrasound testing for one of the three autosomal trisomies or for a thickened nuchal translucency [8]. This method for detecting SCA is not effective in, except for cases of Turner syndrome presenting with cystic hygromas. In addition to screening for the common fetal aneuploidies, NIPT could be used to identify SCA.

In most published NIPT studies, a hypothesis test has been used based on fetal aneuploidy detection, where the Z-score exceeds a predetermined threshold. Further statistical improvements have enabled the identification of cases with mosaicism and expanded the aneuploidy detection of all 23 pairs of chromosomes [9]. An algorithm known as Fetal Copy Number Analysis through Maternal Plasma Sequencing (FCAPS) was further developed to detect close to 100% of ≥10 Mb deletions/duplications without the need to increase the sequencing depth [10].

However, the detection rate of NIPT for SCAs is worse, with a higher false positive rate, than those for other trisomies, particularly for monosomy X. Potential contributions to this high false-positive rate include sequencing bias of guanine and cytosine, maternal mosaicism, maternal XXX, the biological loss of an X chromosome in ageing women, and placental mosaicism [11]. In a previous study with massively parallel genomic sequencing, a positive predictive value (PPV) SCAs of 48.4% a negative predictive value of 100% were reported [12]. Another investigation using the same sequencing technique found a PPV of 54.17% for fetal SCAs [13]. Several factors may have contributed to the relatively low PPV value of MPS-based testing results for SCA as compared with those for more common autosomal trisomies [14–17].

A detection rate for SCA of 96.2% with a false-positive rate of 0.3% was reported earlier [18]. However, most of the previously published data were based on experimental data or such collected as a well-designed study. Reports on NIPT performance of SCA based on real clinical data are still limited. It might be potentially valuable to identify inconsistencies between phenotype and genotype gender, and increase the positive and false positive screening rate of NIPT and the complexity of prenatal counseling, which may narrow the advantages of the new screening method [19].

In the present study, we assessed the accuracy of cfDNA testing based on low-level whole-genome sequencing to screen for fetal SCAs and evaluate the clinical performance of NIPT using cfDNA in maternal plasma in 12,243 consecutive cases from a single center.

## Methods

### Patients and sample collection

The center accepted the referral of any pregnant woman for NIPT, regardless of whether they had undergone previous Down syndrome screening tests. All subjects had a pretest ultrasound scan to ascertain the number of fetuses and gestational age, and to exclude major structural abnormalities. According to the NIPT technical rule issued by National Heath Commission of the People’s Republic of China, the appropriate gestational weeks for fetal free DNA detection in maternal peripheral blood were 12^+ 0^ weeks to 22^+ 6^ weeks. The test protocol required pregnancy within 12^+ 0^ to 20^+ 6^ weeks of gestation and singleton pregnancies. All patients underwent pretest counseling and informed consent was received, and analysis of other chromosomes was also conducted, as previously described.

Five milliliters of maternal peripheral blood were collected into a blood tube containing EDTA. The blood sample was immediately placed for storage at 4 °C until further use. Plasma was prepared within 4 h after the collection (subsequently extended to 8 h) using a two-step centrifugation protocol. The whole-blood sample was first centrifuged at 1600 g for 10 min at 4 °C. The supernatant was transferred to sterile 2.0 ml Eppendorf (EP) tubes placed on ice, which was centrifuged again at 16,000 g for 10 min at 4 °C. The final supernatant was transferred to new EP tubes, which were temporarily stored or transported in dry ice or at − 20 °C if DNA extraction was not performed immediately. Each plasma sample was frozen and thawed only once.

These samples were sent to the NIPT laboratory and processed as ordinary clinical samples. The NIPT and chromosomal analysis from amniocentesis culture was performed independently by two different laboratories, each of which was blinded to the results of the other laboratory. All subsequent molecular tests, including cell-free DNA isolation, library construction and DNA sequencing, and bioinformatics analysis were performed as previously described [20].

### DNA sequencing and bioinformatics analysis

The DNA extraction, library construction, and sequencing were conducted according to the standard protocol of the laboratory of Prenatal Diagnostic Center, Obstetrics and Gynecology Hospital, Capital Medical University Genetics Guidelines. The archived samples were also denoted and recorded by a barcode tracking system and delivered to the NIPT laboratory. A volume of 200 μL of maternal plasma was used for cfDNA extraction by BGISP-300 (BGI, Shenzhen, China) with a nucleic acid extraction kit (BGI, Shenzhen, China).

After DNA extraction, end-repair was carried out by adding end-repair enzymes under the following cycle conditions: 37 °C for 10 min and 65 °C for 15 min, followed by adaptor ligation with label-adaptor and ligase at 23 °C for 20 min. After the end-repair and adaptor ligation, PCR was used to amplify DNA to the desired concentration under the following cycle conditions: 98 °C for 2 min, then 12 cycles at 98 °C for 15 s, 56 °C for 15 s, and 72 °C for 30 s, with a final extension at 72 °C for 5 min. The DNA amplification products were quantified by Qubit® 2.0 (Life Tech, Invitrogen, Carlsbad, CA, USA) using Qubit™ dsDNA HS Assay Kits (Life Tech, Invitrogen, USA) and the concentration ≥ 2 ng/μL was regarded as qualified standards. The volume was calculated according to the concentration of each sample, and each sample of the same mass was mixed by pooling. Fetal chromosome aneuploidies (T21, T18, and T13) detection kit (Combinatorial Probe-Anchor Synthesis Sequencing Method, CPAS) (BGI, Shenzhen, China) was used for library construction.

Double-strand DNA was thermally denatured into single-strand after pooling, followed by the addition of cyclic buffer and ligase to create DNA circles by cyclization. Qualified DNA circles were used to make DNA Nanoballs (DNBs) by rolling-circle replication. The concentration of DNBs was quantified by Qubit® 2.0 using Qubit™ ssDNA assay kits (Life Tech, Invitrogen, USA), and DNBs concentrations within the range 8—40 ng/μL were considered appropriate. Then, DNBs were loaded onto chips and sequenced on a BGISEQ-500 sequencing platform (BGI, Shenzhen, China). Any sample that failed to meet the quality control criteria was reported as detection failure by NIPT.

The average depth of sequencing of this NIPT method for each sample was approximately 0.1X. A minimal number of unique sequencing reads of no less than 3.5 million per sample was obtained using the CPAS sequencer for analysis of chromosomal aneuploidy. A binary hypothesis Z-score was applied to classify aneuploidy. The Z-score obeyed standard normal distribution, within the range 0.001–0.999 (from − 3 to 3), as the threshold of chromosomal aneuploidy. Z-score was set within the range from − 3 to 3 as the threshold to evaluate the risk of chromosomal aneuploidies. If Z-score > 3 or Z-score < − 3, the sample was classified to be with a high risk of chromosomal aneuploidies. If the Z-score was between − 3 and 3, the sample was classified as having a low risk.

### Results reporting

After the MPS-based test, all participants were given a routine test report showing the estimated fetal risk (positive or negative) of trisomies 13, 18, and 21, and the suspected risk of SCA was reported to the clinician in the form of a supplementary report. All patients were informed for the possibility of such an additional finding and given appropriate counseling. Fetal sex was not reported, even on request, unless sex-chromosomal abnormalities were suspected.

A follow-up including confirmatory invasive testing was recommended for all positive results for trisomies 21, 18, and 13. The subsequent management of other suspected abnormalities depended on individualized counseling results.

### Confirmatory invasive testing

Patients identified with SCA-associated pregnancies were offered to undergo prenatal fetal chromosomal karyotyping according to International System for Human Cytogenomic Nomenclature (ISCN 2016) [21]. Conventional G-banded cytogenetic studies were performed in the laboratory of the Prenatal Diagnostic Center, Obstetrics and Gynecology Hospital, Capital Medical University. Prenatal samples were cultured following previously described standard protocols [22]. At least 20 G-banded metaphases from each sample were analyzed using the Wright’s staining method [23].

For invasive testing, we employed liquid microarray detection hybridization by bacterial artificial chromosome (BAC) for rapid analysis and harrumphing. BACs-on-Beads (BOBs) microarray detection is a liquid chip-based analytical technique that simultaneously hybridizes biotin-labeled sample DNA and reference DNA to microspheres that have a specific BACs DNA probe, immobilized using Luminex. The system performs microsphere classification and fluorescence signal detection, and the results are interpreted by the software of BOBs to detect abnormal chromosome numbers and microdeletions.

A negative NIPT was confirmed if prenatal karyotyping was normal. The pregnant woman was then allowed to make her own decision to continue or terminate the pregnancy. Placental tissues were not obtained in any of the NIPT SCA-positive cases.

### Follow-up of pregnancy outcome information

Pregnancy outcome information was collected from referral doctors and prenatal and postnatal laboratory results. Patients were contacted regularly for understanding the fetal outcome until either pregnancy termination or delivery. The outcome of the high-risk cases was specifically followed up. A failure of contact was declared after three or more unsuccessful attempts to contact a patient.

## Results

From March 2018 to February 2019, a total number of 12,243 singleton pregnant women in our cohort agreed to undergo cfDNA screening with available NIPT results, whose gestational age at blood sampling was within 12–20 weeks. Of these, 3932 pregnant women agreed to prenatal biochemical screening prior to NIPT, and 8311 chose to undergo NIPT directly during the second trimester. The 12,243 pregnant women were all of Chinese ethnic background with a mean ± SD maternal age of 33 ± 4 years (~ 40% were 35 years old or older), which included 610 pregnancies that were in vitro fertilized (IVF) (Table 1). Overall, the positive aneuploidy screening rate was 1.38% (169/12243). High risk results were recorded for SCA in 44 women (0.359%), including one with dual aneuploidy (trisomy 18 and XXX).

**Table 1:** Demographic and clinical characteristics of the pregnant women (*n* = 12,243) enrolled in a study that investigated the use of noninvasive prenatal testing (NIPT) for screening for fetal sex chromosome aneuploidies

Characteristic	n (%)
Chinese	*12,243*
Singleton pregnancy	12,243
Gestational age at NIPT 12–20 weeks	12,243
Routine prenatal screening results	3932(32.12)
High risk	716(5.85)
Intermediate risk	2903(23.71)
Low risk	313(2.56)
Others	8311(67.88)
IVF	610(4.98)
Maternal age, years	
<35	7382(60.30)
≧35	4861(39.70)
Total NIPT screen-positive	169(1.38)

*Abbreviation*: *IVF* In vitro fertilization

NIPT screening yielded 44 results positive for SCA, including twenty-one positive MPS results for 45,X, six for XXX, twelve for XXY, and five for XYY. The mean ± SD maternal age of the 44 patients with NIPT SCA-positive results was 34 ± 4 years (17 cases were 35 years old or older). Thirty-two of the 44 women chose to undergo invasive prenatal diagnosis, but one miscarried before its amniocentesis, whereas eleven cases (25.0%) declined the invasive procedure (Table 2). Prenatal karyotyping and BOBs, both from amniocentesis, were performed in eleven cases, remaining 16 and 6 of the SCA-positive cases, respectively, in which only either karyotyping or BOBs was conducted. The participant with dual aneuploidy high risk results (trisomy 18 and XXX) made the decision on invasive prenatal care based on the autosomal abnormality rather than the SCA, and her data were also included in the SCA invasive testing results discussed here.

**Table 2:** Karyotyping in screening positive sex chromosome abnormality cases

SCA type	Screening positive, n	BOBs	Karyotype	Total (%)	declined
45,X	21	6	11**a**	14(66.7)	7
47,XXX	6	2	3	4(66.7)**b**	2**c**
47,XXY	12	8	9	11(91.7)	1
47,XYY	5	1	4	4(80.0)	1
All SCAs	*44*	17c	27	33(75.0)	11(25.0)

Thirty-two of the 44 patients underwent further karyotype analysis, besides one pregnant woman who had a miscarriage underwent a postnatal diagnosis of the abortion tissue. The karyotype information is not available in 11 screening positive SCA cases which were declined

a-A screen-positive 45,X pregnant woman miscarriage and a karyotype analysis using abortion tissue found to be 45,X

b-One case of dual aneuploidy (trisomy 18/XXX) included where invasive prenatal testing decisions was dictated by the autosomal aneuploidy component of the result

c-One case was decline and one case selected the peripheral blood karyotype analysis since maternal abnormality which is 47,XXX

d-Three 45,X, one 47,XXX and two 47,XYY have chosen to invasive prenatal testing via BOBs rather than karyotyping

Of the 33 pregnant SCA-positive women, karyotype results confirmed SCA in 19 cases (three cases of 45,X, three of 47,XXX, ten of 47,XXY, and three of XYY), whereas 14 cases had a normal karyotype (Table 3). The overall PPV (i.e., the chance of a positive result being confirmed by diagnostic testing) of all NIPT-positive results for fetal SCA was 57.6% (19/33), the lowest individual PPV was observed for monosomy X at 21.4% (3/14). Interestingly, one of the XXY-positive cases diagnosed by NIPT with a negative confirmation obtained by invasive testing was found to contain X chromosomal microdeletions 46,X,del(X)(p22.2). Because placental tissues were not obtained from the 44 NIPT SCA-positive cases, the possibility of placental mosaicism influencing false-positive results could not be assessed.

**Table 3:** Non-invasive prenatal testing performance for detection of fetal sex chromosome aneuploidy type

SCA type	Screening positive, n	True-positive results	False-positive results	Unknown karyotype	PPV, %	TOP
45,X	21	3	11	7	3/14(21.4)	2
47,XXX	6	3a	1	2	3/4(75.0)	3
47,XXY	12	10b	1	1	10/11(90.9)	11**c**
47,XYY	5	3d	1	1	3/4(75.0)	3
All SCAs	44	19	14	11	19/33(57.6)	19

Of those with positive screening results for sex chromosome abnormalities, karyotypic information was available in 33 cases, and the PPV, positive predictive value of a prenatally confirmed SCA after an invasive test

a-Includes a case at high risk for both trisomy 18 and XXX (true-positive for both)

b-One Mosaic of 47,XXY[96]/46,XY[4]

c-One 47,XXY case of negative confirmation by karyotype found to be X chromosomal microdeletions has chosen termination of pregnancy too

d-One Mosaic of 47,XXY[35]/46,XY[51]

*Abbreviation*: *TOP* Termination of pregnancy

Of 14 screening-positive 45,X cases that underwent karyotyping, two were prenatally confirmed to have SCA after an invasive test as 45,X; they choose to terminate their pregnancies (Table 4). In addition, one 45,X karyotype was confirmed on products of conception following a spontaneous abortion, due to Turner syndrome. One of eleven normal karyotypes cases showed 46,XY but the patient miscarried for unknown reasons. The remaining ten 46,XX patients decided to continue their pregnancies and delivered phenotypically healthy babies.

**Table 4:** Findings in Pregnancies with SCAs Predicted by NIPT

Case	SCA	Maternal Age	Routine Screen texts	BOBs	Fetal Karyotype	Maternal Karyotype	Pregnancy Outcome
1	45,X	36	Normal	ND	46,XX	ND	Normal female
2	45,X	32	T21 1/996	46,XX	46,XX	ND	Normal female
3	45,X	27	NT 2.7 mm	45,X	ND	ND	TOP
4	45,X	38	Normal	ND	46,XY	ND	Miscarriage
5	45,X	34	Normal	ND	46,XX	46,XX	Normal female
6	45,X	31	T21 1/615	46,XX	46,XX	ND	Normal female
7	45,X	29	T21 1/895	Declined	Declined	ND	Unknown
8	45,X	35	Normal	ND	46,XX	ND	Normal female
9	45,X	34	Normal	Declined	Declined	ND	Unknown
10	45,X	30	NT 5.4 mm pleural effusion	ND	45,X	ND	TOP
11	45,X	36	Normal	Declined	Declined	ND	Unknown
12	45,X	30	NT 7.7 mm	ND	ND	ND	Unknown
13	45,X	31	T21 1/424	ND	ND	ND	Unknown
14	45,X	27	T21 1/252	ND	POC 45,X	ND	Miscarriage
15	45,X	31	T21 1/758	ND	46,XX	ND	Unknown
16	45,X	33	hypertension	46,XX	ND	ND	Unknown
17	45,X	32	Normal	Declined	Declined	ND	Unknown
18	45,X	39	Normal	Declined	Declined	ND	Unknown
19	45,X	34	NT 2.1 mm	46,XX	ND	ND	Unknown
20	45,X	34	Normal	ND	46,XX	ND	Unknown
21	45,X	33	T21 1/911	46,XX	46,XX	ND	Unknown
22	XXX	37	Suspected XXX	Declined	Declined	47,XXX	Normal female
23	XXX/T18	35	Normal	mos 47,XXX, dup(18)(q12–22)/47,XXX	mos 47,XXX[74]/47,XX,+ 18[26]	ND	TOP
24	XXX	36	Normal	Declined	Declined	ND	Normal female
25	XXX	33	T21 1/460	ND	46,XY	ND	Normal male
26	XXX	34	Normal	ND	47,XXX	ND	TOP
27	XXX	28	T21 1/429 NT 2.4 mm	47,XXX	ND	ND	TOP
28	XXY	34	Normal	ND	47,XXY	ND	TOP
29	XXY	37	Normal	ND	47,XXY	ND	TOP
30	XXY	39	Fetal death history	47,XXY	47,XXY	ND	TOP
31	XXY	36	Normal	Normal	46,X,del(X)(p22.2)	ND	TOP
32	XXY	44	Normal	47,XXY	47,XXY	ND	TOP
33	XXY	36	Normal	47,XXY	47,XXY	ND	TOP
34	XXY	22	T21 1/460	47,XXY	47,XXY[96]/46,XY[4]	ND	TOP
35	XXY	37	Normal	47,XXY	47,XXY	ND	TOP
36	XXY	35	Normal	47,XXY	ND	ND	TOP
37	XXY	25	Fetal death history	ND	Declined	ND	Unknown
38	XXY	26	Normal	ND	47,XXY	ND	TOP
39	XXY	34	Normal	47,XXY	ND	ND	TOP
40	XYY	35	Fetal death history	Declined	Declined	ND	Normal male
41	XYY	34	Normal	XXY/XY	47,XXY[35]/46,XY[51]	ND	TOP
42	XYY	31	T21 1/926	ND	46,XX	ND	Normal female
43	XYY	38	Hypertension	ND	47,XYY	ND	TOP
44	XYY	34	NT 3.8 mm	ND	47,XYY	ND	TOP

*Abbreviations*: *NT* Nuchal translucency, *ND* Not done, *POC* Products of conception

Of the four screening-positive XXX cases subjected to karyotyping, two were confirmed as 47,XXX karyotype and one with mos 47,XXX[74]/47,XX,+ 18[26] karyotype, who terminated the pregnancy, whereas the false-positive one had normal karyotype 46,XY (Table 4). Moreover, another woman chose karyotyping though peripheral blood rather than amniocentesis due to abnormal performance herself, which indicated it was due to maternal SCA (47,XXX). Although no more tissues were analyzed, the women decided to continue the pregnancy and delivered phenotypically healthy babies.

Eleven karyotyping results were obtained in the twelve screening-positive XXY group, ten of which were concordant with the NIPT results including one with mos 47,XXY[96]/46,XY[4] karyotype. The false-positive one had X chromosomal microdeletions with the 46,X,del(X)(p22.2) karyotype, which caused a X-link disease, linear skin defects with multiple congenital anomalies 1 (LSDMCA1), also known as microphthalmia with linear skin defects (MLS, OMIM 309801), the patient chose to terminate the pregnancy carefully (Table 4).

Three cases terminated pregnancies based on karyotyping in the screening-positive XYY patients, and two cases were confirmed 47,XYY and another fetal with karyotype mos 47,XXY[35]/46,XY[51]. In addition, one XYY case had a normal karyotype 46,XX and one case declined prenatal diagnosis. The pregnant women with the XXX/XYY-type, who had declined prenatal diagnosis, delivered three phenotypically normal neonates (Table 4).

## Discussion

Cell-free fetal DNA analysis of maternal blood investigations cannot be applied only for the screening of trisomies 21, 18, and 13, but is also potentially applicable for the detection of other aneuploidies, including sex chromosome ones. However, in our study, the detection rates were different from those of previous examinations, and thus using positive predictive values as a measure is a more accurate approach. The detection rate of SCA can only be accurately assessed if postnatal karyotyping is performed in every case. Here, seven NIPT-tested 45,X high-risk women declined a prenatal diagnosis and we did not obtain follow-up results on whether the karyotype was actually 45,X. Hence, PPV would have been 47.6% (10/21), and the lower limit PPV of 45,X would have been 14.3% (3/21), if these 7 patients were regarded as false positive (Table 2).

Cell-free fetal DNA originates mainly from placental trophoblasts, which are often mosaic. Thus, the selection of diagnostic testing after a high-risk SCA result also requires a careful consideration. Amniocentesis allows for a more accurate display of the true fetal karyotype with a lower potential for mosaicism, whereas chorionic villus sampling still shows the placental components. However, a more informative result from amniocentesis can potentially require a one-month period of waiting, which increases the risk of pregnancy loss. Ultrasound screening in a large population achieved nuchal translucency (NT) above the 95th centile in 88% of 33 fetuses diagnosed with monosomy X, and in 40% of 20 fetuses with other SCA [24]. In the true positives in our population, two cases were detected by fetal enlarged nuchal translucency by ultrasound, and pleural effusion was detected in one of them. On the other hand, one 45,X-confirmed case chose NIPT not in the setting of enlarged NT in our cohort, which contradicts the benefits of continuing NT assessment even when NIPT is performed. Nonetheless, NT measurements may help to distinguish true positive from false positive monosomy-X NIPT screening results.

Circulating cell-free DNA is derived from both maternal and placental tissues, so intrinsic biological factors, such as maternal or fetal mosaicism and undiagnosed maternal SCA (one case in our series) frequently have an influence on NIPT accuracy [25–28]. Hence, it is possible that the SCA-positive cases were caused by fetal mosaicism, which would be difficult to confirm by traditional karyotyping if insufficient cells or tissues were analyzed. To avoid this from happening, we carried out both BOBs and karyotype analysis in most cases. It was found that three mosaic in our series by invasive prenatal diagnosis, but we didn’t have sufficient samples to distinguish which mosaicism type they are.

The limited placental mosaicism or maternal mosaicism can lead to SCA-positive results, in which the degree of mosaicism will impact the performance of the test because it will interfere with the effective fetal fraction. This can be confirmed by further analysis of placental tissue or maternal white blood cells [28–30]. However, placental tissues were not obtained in this study. Low levels of mosaicism for sex-chromosome aneuploidy in general, monosomy-X in particular, can be present in apparently healthy women [31, 32]. Ideally, all maternal karyotypes in these cases should be identified, but this was not routinely available at the time of conducting this study. Therefore, this potential for mosaicism should be considered as a limitation of our NIPT assay.

Additionally, in our population, one sample had a positive NIPT-based XXX result, and one had 45,X but prenatally confirmed 46,XY karyotype on amniocentesis, and a XYY-positive NIPT finding provably showed 46,XX karyotype. We did not consider the phenomenon of chimerism due to twin fusion or vanishing twin, as we chose singleton pregnancies confirmed by ultrasound from the beginning. This phenomenon may be partially explained by the lack of the unique genes on the Y chromosome, which is known as the pseudo-autosomal region on the X chromosome. There was still one XXY-positive on NIPT that was detected as false-positive by BOBs and a microdeletion on X chromosome found after karyotyping. This discordance might have been caused by inaccurate diagnoses of X-chromosome aneuploidies, since 58 genes were homologous on two sex chromosomes the majority of which (29 genes) are located at the ends of the X and Y chromosomes. Potential contributions to this discordant false-positive rate which was documented included sequencing bias of guanine and cytosine on the X chromosome, which, along with the phenomenon of the loss of X chromosome in aging women, requires further investigation [18, 33].

The limitations of our study were its small size and our inability to determine false negative rates for NIPT detection of SCA due to the difficulties in the screening of each newborn baby by karyotyping. Neonates with SCA can appear normally, without physical or intellectual disability, and thus it is difficult to identify SCA syndrome before puberty without karyotyping. Furthermore, no phenotype was determined at birth in three XXX/XYY cases, which might have also been SCA-positive. Thus, postnatal diagnosis is strongly recommended. In addition, a relatively small number of pregnant women from a single center were enrolled in the study, which had an incidence of approximately 1 in 500 newborns. Larger multicenter studies are warranted to corroborate these finding.

## Conclusions

NIPT can identify fetal SCAs by screening cfDNA from the mother’s plasma using massively parallel genomic sequencing, while the accuracy needs to be improved, especially for Turner syndrome. Although the overall PPV (defined as number of prenatally confirmed SCA cases as a percentage of all screen-positive results for SCA) was only 43.2% (19/44), at least 19 pregnant women, including the microdeletion samples, were identified and intervened in our cohort. We detected not only abnormal numbers of sex chromosomes, but also structural abnormalities. Therefore, we suggest that all NIPT screen-positive subjects should be carefully counseled about the potential contribution of the fetal, placental or maternal compartment.

## Data Availability

The datasets used and/or analyzed during the current study are available from the corresponding author on reasonable request.
